# Annotations

**Published:** 1894-11-17

**Authors:** 


### ANNOTATIONS.

### Text-Book Surgery.

It is always a matter of interest, and is strongly suggestive of progress, when the living surgery of the societies is in opposition to the more crystalised surgery of the text-books. An instance of this kind occurred at the Medical Society on Monday, when Mr. Clutton showed a boy who, having been run over by a waggon, had suffered from fracture of many ribs, dislocation of the shoulder, and fracture of the surgical neck of the humerus. The patient on admission was very ill, the lung collapsed, and the skin emphysematous from hip to clavicle. It was impossible at first to do anything to the shoulder, and when an attempt was made, under chloroform, to reduce the dislocation every effort to do so was found unavailing. The capsule of the joint was therefore opened, the head of the bone replaced, the upper end of the shaft, which was separated by capsule from the head, restored to its position, and the fragments pinned together by an ivory peg passed through the head downwards into the shaft. The result as shown to the society was excellent, very perfect mobility in every direction having been obtained. Mr. Clutton remarked that all the text-books advise such a complication to be treated by first dealing with the fracture, and when union has been effected then attempting the reduction of the dislocation, a manœuvre which, he said, generally resulted in the bone giving way at the seat of fracture, the second state being worse than the first. Clearly modern surgery hinges on the absolute confidence which surgeons have in their power of avoiding suppuration. That once being granted the mild and safe counsels of the text-books sink to the level of respectable platitudes. All the same these platitudes are useful axioms for mediocre men, for no man has a right to practice modern surgery till he is sure of all the details of the modern methods on which its safety and success depend.

### Glasgow's Hospital Sunday.

Glasgow is rich and, in some directions, very generous. She is specially generous towards her ecclesiastical institutions, of which she possesses an extraordinary number and variety. Of a well-known ornate church at the west end of the city it is said that on its dedication day some wag had chalked up the following couplet in large letters on one of the outer walls: "This church is not for the poor and needy, but for the rich and Dr. Eady." But though so generous in directions ecclesiastical, Glasgow does not appear to place the Good Samaritan on the same level as the Priest and the Levite in her appreciation. Last Sunday the western city made her first Hospital Sunday collections, 265 congregations and 184 Sunday schools joining in this great annual effort. The total amount collected by all these churches and Sunday-schools was £3,210 14s. 8d. Now Glasgow claims, not without show of reason, to be the "second city in the Empire." Another city, Liverpool, also makes claim to be the "second city." It cannot but be of interest to the people of Glasgow to know how Liverpool, Conservative and Episcopal Liverpool, compares with their Radical and Presbyterian city in the matter of Hospital Sunday. Liverpool collects on an average close upon £7,000 annually on her Hospital Sunday; a good deal more than twice as much as her rival "second city." Besides this, the workshop and Saturday collections of Liverpool amount to the handsome total of £4,000 to £4,500 every year. There is food for rumination here for the proud and not unthoughtful citizens of the Scottish city. Some of the congregations, particularly that of the Park Church, whose minister is the Rev. Dr. Donald McLeod, have given with considerable generosity, though even its giving compares but poorly with that of several of the best known London churches. Other congregations, notably that of St. Matthew's Free Church, of which the Rev. Dr. Stalker is the minister, have greatly disappointed expectation. The miserable total of £41 5s. 3d. for such a congregation almost makes one blush in London, and several days after the event. A city which can raise £10,000 by a single bazaar, as Glasgow has recently done two years in succession for charitable objects, shows an extraordinary want of judgment in its benevolence when a general effort of all its congregations cannot get beyond the melancholy total of £3,210 14s. 8d. for all its hospitals. Where is the fault? No doubt it lies in the lack of effective organisation. "Organisation and simultaneity." These must be the watchwords of Glasgow's next Hospital Sunday collection if she is to show a result worthy at once of her reputation for well-considered benevolence and of her proud position in the great empire of which she is so considerable a part.

### "Cures" for Drunkenness.

A "Perplexed" Hibernian is "very anxious to know if there be a cure for drunkenness," and where he may apply for information. In a matter of this kind nothing is so valuable as actual experience, and this we can place at "Perplexed's" service. There is an entry in a certain physician's case book, bearing date December 15th, 1893. The entry states that about September, 1892, a patient underwent the gold cure at Winnipeg, which, if not the actual place of origin of the "gold cure," is at least very near that spot. The patient treated related his own experiences to the physician from whose case book we are quoting. A preparation of gold was, he said, injected into his upper arm, and after producing many symptoms of a most unpleasant kind, ultimately made him feel "as if he were going to be a raving lunatic." The net result was, to use his own words, that in his case "the gold cure was a fraud." He also volunteered this statement of fact, which was taken down at the time from his own lips, and which any curious person is at liberty to read on showing cause. In all, he knew of "twelve cases treated. All, or most of these, seemed to be better for six or eight months. But he was quite certain that in the final result all, or most of them, became worse drunkards than before." In our judgment, the "cures" for drunkenness which are now being so freely advertised are like the many vaunted "cures" for cancer, frauds, pure and simple. It is one of the easiest things in the whole range of physic to nauseate a man so that he will hate the taste of drink, but when the nauseating drug is withdrawn the drink appetite returns. The only real cure for drunkenness is complete isolation for a sufficient length of time, with moral suasion, and the building up of the general health.

